# Non-muscle myosin II as a predictive factor in head and neck squamous cell carcinoma

**DOI:** 10.4317/medoral.22898

**Published:** 2019-04-24

**Authors:** Kelly-Bienk Dias, Anacláudia-Pereira-Costa Flores, Laura-de Campos Hildebrand, Márcia-Gaiger de Oliveira, Marcelo-Lazzaron Lamers, Pantelis-Varvaki Rados, Alessandra-Selinger Magnusson, Manoel-Sant’Ana Filho

**Affiliations:** 1 DDS, Msc., PhD student - Oral Pathology - Universidade Federal do Rio Grande do Sul - School of Dentistry; 2 DDS, PhD. Assistant Professor - Oral Pathology - Universidade Federal do Rio Grande do Sul – School of Dentistry; 3 DDS, PhD. Assistant Professor - Basic Research Center - Universidade Federal do Rio Grande do Sul - School of Dentistry; 4 DDS, PhD. Titular Professor - Oral Pathology - Universidade Federal do Rio Grande do Sul – School of Dentistry; 5 BPharm. Pharmacist - Oral Pathology - Universidade Federal do Rio Grande do Sul – School of Dentistry

## Abstract

**Background:**

The present study attempted to provide information regarding non-muscle myosin II (MII) isoforms immunoreactivity in patients with head and neck squamous cell carcinoma (HNSCC) and analysis of the patients’ clinical status after 5 years of monitoring.

**Material and Methods:**

A semiquantitative analysis of the immunoreactivity of the MII isoforms was performed in 54 surgical specimens and its correlation with clinical and pathological variables and prognosis was verified. Data were analyzed using chi-square, Mann-Whitney and Kruskal-Wallis tests. To evaluate the survival over the total monitoring time and any connection with the proteins studied, the Kaplan-Meier analysis was used. *P* values ≤0.05 were considered statistically significant.

**Results:**

In the advanced stages of pathological tumor-node-metastasis, the expression of MIIB in adjacent non-neoplastic epithelial tissues tended to increase (*p* = 0.057). In tumoral zones there was an association of high expression among the three isoforms (MIIA/MIIB *p*=0,001, MIIB/MIIC *p*=0,006 and MIIA/MIIC *p*=0,012). Negative clinical evolution in patients was directly correlated to increased MIIC expression in the tumoral zone of invasion in HNSCC (*p* = 0.017). Based on clinical evolution after the monitoring period, patients with tumors expressing MIIC had poorer prognoses (*p* = 0.048).

**Conclusions:**

The present study suggests that MIIB expression in non-neoplastic adjacent epithelial tissues may indicate a potential for regional metastasis and that MIIC expression in the tumoral zone of invasion is predictive of negative evolution of the disease.

** Key words:**Head and neck squamous cell carcinoma, oral cancer, myosin type II, non-muscle myosin, immunohistochemistry.

## Introduction

Squamous cell carcinoma, a disease linked to risk factors such as tobacco and alcohol consumption, is the most common malignant neoplasm of the head and neck (HNSCC). In addition to these major risk factors, sun exposure, eating habits and types 16 and 18 of human papillomavirus (HPV) are related to the pathogenesis of the disease, mostly in the oropharynx (1). Despite advancements in treatment, the mortality rate of HNSCC continues to be high and the survival rate low (2). Tools for provision of prognostic information for patients suffering from malignant neoplasia, such as histological grading and the TNM system, are questioned by some studies since a solid tumor such as HNSCC presents an uneven cellular population, resulting in different behaviors in distinct tumor zones, often masking the real degree of progression of the disease (3). The causes of treatment failure and poor prognosis include tissue invasion and development of metastatic potential, which is regulated by molecular alterations such as modifications to cellular adhesion to the extracellular environment, allowing displacement of the cell, an indispensable process for tumor progression (4,5). Among the molecules involved in cellular locomotion, type II non-muscle myosin (MII) plays a key role in regulation of different migratory events through generation of contractile forces that align actin filaments and control adhesion maturation, nucleus displacement, and cellular body retraction during cytokinesis (3,5). The present study attempted to provide information concerning the immunohistochemical expression of MII isoforms (A, B, and C) in patients with HNSCC through evaluation of MII immunoreactivity in surgical specimens, and analysis of patient clinical status after 5 years of monitoring.

## Material and Methods

-Description of the Sample

This sample is composed by 54 patients included in a previous study approved by the Research Ethics Committee of the Porto Alegre Clinical Hospital (HCPA) on August 28, 2009 (09-315). The collection lasted twelve months from October 2009 and was composed by patients diagnosed with HNSCC and treated at the Surgery Ambulatory of Head and Neck in the HCPA. All individuals were older than 18 years at the time of diagnosis, and they each signed an informed consent form as well as answered a survey used to obtain demographic data such as age, gender and deleterious habits including tobacco and alcohol. Paraffin blocks were prepared with the HNSCC samples and the tumor clinical data was performed at the time of surgery (6). After this, these patients were followed up until July 2015.

-Evaluation of Tumor Samples

**Figure 1:**
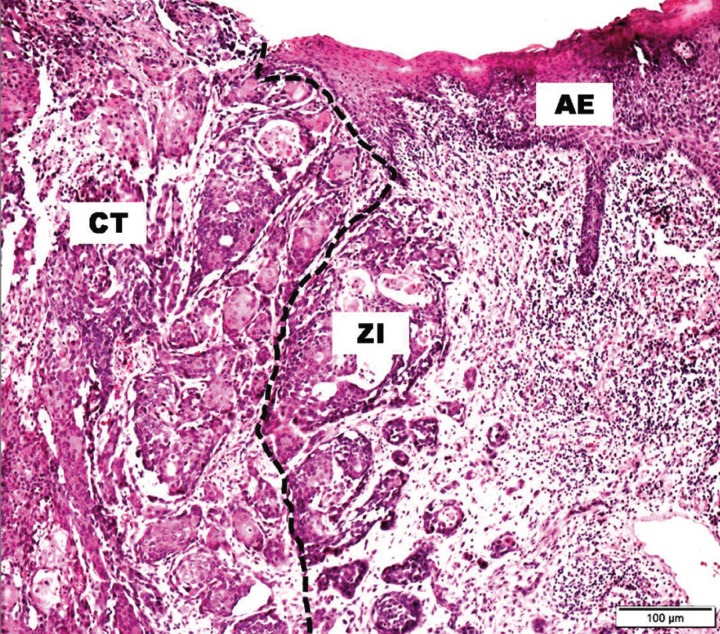
Representative hematoxylin and eosin staining of the HNSCC center of the tumor (CT), zone of invasion (ZI), and adjacent non-neoplastic epithelial tissues (AE). Original magnification 200×. SOURCE: Oral Pathology FO/UFRGS.

Histological cuts of 3 µm were obtained from the paraffin blocks prepared earlier (6) and stained with hematoxylin and eosin. For each HNSCC sample, tumoral zones were identified as the center of the tumor (CT) and the zone of invasion (ZI), as well as non-neoplastic adjacent epithelial tissues (AE) (7) (Fig. 1).

Histological grading was performed according to the criteria of Bryne *et al.* (8) by two pathologists (A.P.C.F and K.B.D), in which a score was attributed for each morphological characteristic being evaluated, and individual scores were combined to determine to which group of malignancy the specimen belonged: low, moderate, or high.

-Clinical Staging

Clinical data of the tumors, such as tumor size (T), node status (N), and the presence of metastases (M),were tabulated and classified according to the TNM system, following the parameters established by The American Joint Committee on Cancer, during the examination of the surgical specimens (pTNM) (9). The outcome of 5 years of follow-up was classified as failure (local recurrence, regional or distant metastasis and death due to tumor) or disease-free survival (7).

-Immunohistochemistry

Histological sections of 3 µm were made using silanized slides for immunostaining of the MII isoforms (A, B, and C). Initially, the cuts were deparaffinized in xylol, rehydrated in a gradient of alcohol and water, and immersed in 3% hydrogen peroxide solution to block the endogenous peroxidase activity. Afterwards, they were submitted to antigen retrieval and incubated with primary antibodies anti-MIIA, anti-MIIB, and anti-MIIC (IIA, polyclonal; IIB, clone D8H8; IIC, clone D4A7; rabbit antibodies; Cell Signaling; 1:50 dilution). Antigen retrieval of the anti-MIIA epitope was conducted by incubation in a pressure cooker at 120°C for 3 minutes and of the anti-MII B and C epitopes by treatment with 0.25% trypsin for 30 minutes. Incubation of all antibodies was conducted at 4°C overnight. A positive control (stomach adenocarcinoma) and a negative control (non-immune serum from the same animal species as the primary antibody) were used according to the manufacturer’s instructions. The detection system used was polymeric (Envision dual link; Dako, Carpentaria, CA, USA), and the samples were developed with a chromogen solution containing 0.03% 3-3,3′-diaminobenzidine (DAB, DakoCytomation, USA). The samples were counterstained with a solution of Mayer’s hematoxylin.

-Data Analysis

Tumoral cells that presented brown cytoplasmic stainingwere considered positive for MIIA, MIIB, and MIIC. The immunostained histological cuts were submitted for semi-quantitative analysis based on the extent of immunoreactivity. Samples were considered positive when more than 30% of the tumor cells were marked. Immunohistochemical analysis was performed by two pathologists (A.P.C.F and K.B.D), and reproducibility was confirmed over the study period by randomly selecting one out of every 10 slides for reassessment after a 7-day interval (kappa > 0.7). During the evaluation, examiners were blinded to the origin of the material.

-Statistical Analysis

Data were analyzed using SPSS version 21. The relationship between the immunostaining of the proteins was evaluated using the chi-squared test; the immunostaining distribution of the proteins in the tumoral zones according to the evolution, and exposure to alcohol and smoke were verified by Mann–Whitney tests. Kruskal–Wallis tests were used to evaluate the distribution of the protein immunostaining in the tumoral zones, according to the histological grading and pTNM system. To evaluate survival over the total time of monitoring and any connection to the studied proteins, Kaplan-Meier analysis was used. *P* values ≤0.05 were considered statistically significant.

## Results

Among the patients evaluated, 85.2% were men and 14.8% women, the mean age was 58.3, and the predominant age group was over 65 years old. The majority of the occurrences of HNSCC were in the mouth (68.5%) in relation to the patients affected in the neck (31.5%) ( Table 1).

**Table 1:**
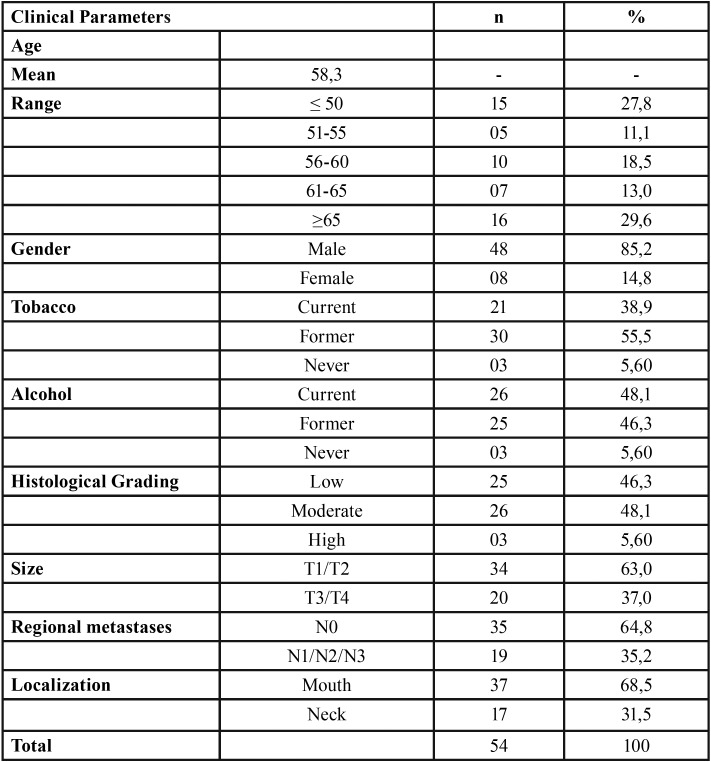
Demographic data and tumor features.

According to the pTNM system, 63% presented tumor sizes between T1 and T2, 64.8% did not present regional metastasis (N0), and there were no distant metastases (MX, M0, and M1) ( Table 1). In advanced pTNM stages, the expression of MIIB in AE tended to increase (*p* = 0.057) ( Table 1).

According to histological grading, 48.1% of the HNSCC samples were of moderate grade, and no statistical correlations between the expression of MIIs in the AE and the tumoral zones were observed ( Table 1).

At the time of the diagnosis, 38.9% of the patients were exposed to tobacco and 48.1% to alcohol ( Table 1). Of the patients exposed to alcohol, 83.3% had maintained their habit for a period longer than 10 years. At an average of 4.7 and 6.3 years before HNSCC diagnosis, 55.5% of the patients had ceased tobacco use and 46.3% had stopped alcohol consumption, respectively.

There was no statistically significant correlation between exposure to tobacco and alcohol with protein expression. However, in patients exposed to alcohol, a correlation with immunostaining for MIIA (*p* = 0.048) and MIIB (*p* = 0.010) was found in the AE ( Table 2).

**Table 2:**
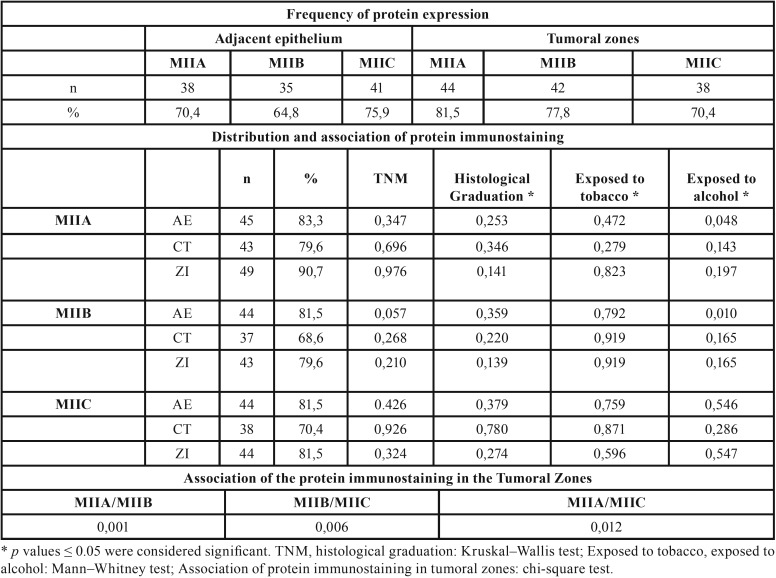
Isoforms expression frequency and their associations.

In tumoral zones, there was an association of high expression among the three isoforms ( Table 2).

**Figure 2:**
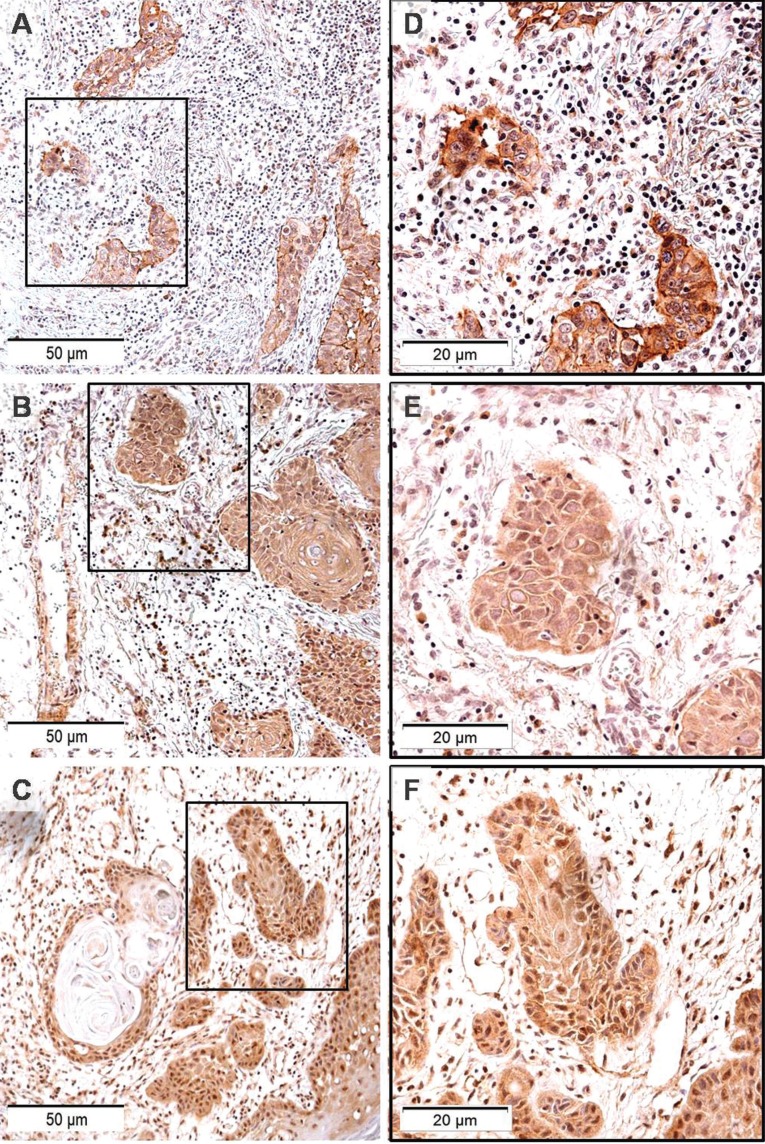
Representative images of MIIC immunolabeling in ZI of HNSCCs: A, B and C original magnification 200×; D, E and F, highlighting cytoplasmic and nuclear marking. Original magnification 400×. SOURCE: Oral Pathology FO/UFRGS.

**Figure 3:**
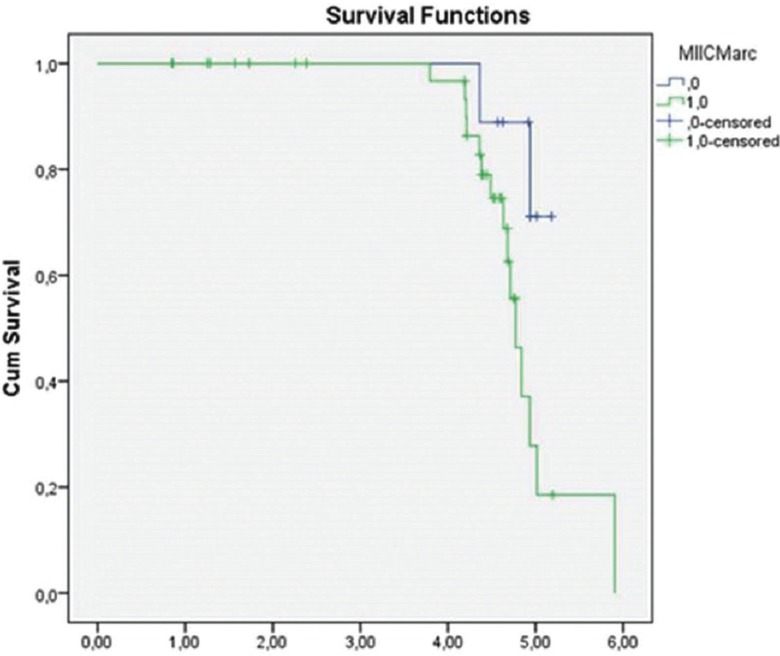
Kaplan-Meier curve showing, according to the time of monitoring, the evolution of survival in patients with tumors showing MIIC expression (0.146, standard deviation, 95% confidence.

The maximum time of monitoring was 5.9 years, and from the total of 54 initial patients, we obtained the final status for 46 patients. Among these patients, 58.1% showed positive evolution (disease free survival) and 41.9% exhibited negative evolution (treatment failure). The average number of years for patients to present failure was 5.01.Negative clinical evolution in patients was directly correlated to increased MIIC expression (*p* = 0.017) ( Table 3) in the ZI of the HNSCC cases (Fig. 2). Based on the evolution after the monitoring period, patients with tumors with MIIC expression had poorer prognosis (*p* = 0.048) (Fig. 3).

**Table 3:**
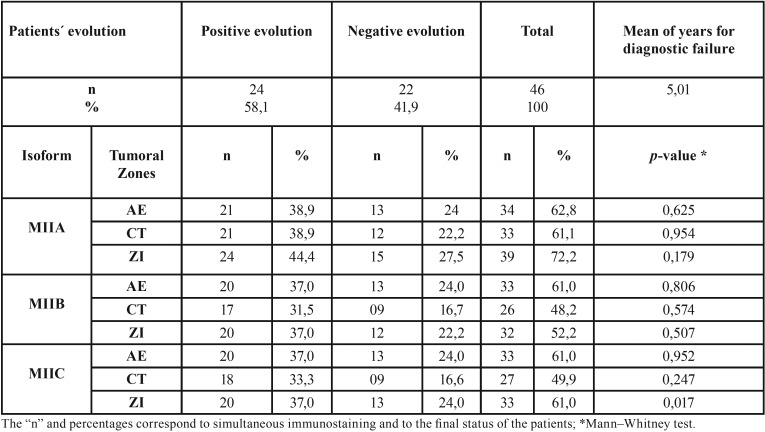
Clinical evolution and its relationship with protein immunostaining.

## Discussion

The mean age of the patients assessed in this study was 58.3 years, while 29.6% of patients were over 65 years old, in agreement with the literature (10). In the patient sample described, 85.2% were men, which correlates well with a 2- to 4-fold higher incidence of male suffering from HNSCC in comparison to female, according to data described by Globocan (11).

The TNM system, although suggested as a useful tool for forming therapeutic decisions, is not indicated for prognostic prediction. This makes it necessary to obtain more prognostic tools, such as markers related to the metastatic process (12). This was confirmed in this study, where the majority of patients were in the early stages (T1/T2 = 63%, N0 = 64.8%) and the evolution rate was highly negative (41.9%). We have to consider that one of the limitations of this study is that at the time of sample collection (2009) the condition of the patients in relation to HPV was not investigated since the HNSCC related to this viral infection predominate in the region of oropharynx and present different clinical, microscopic and molecular features (13). Only in the year 2017 was the new TNM edition published, considering a different staging for HPV positive patients, confirming this condition as a limitation not only of this study but for the staging of the disease itself (14).

Corroborating studies that questioned the value of histological grading as a predictive factor for prognosis (12), only 5.6% of HNSCC samples from this study had high histological grades, and the proportion of patients with good evolution (58.1%) was small.

Analysis of the behavioral profile of tumoral zones is indispensable since a cell population in the ZI of the HNSCC resembles that found in metastases (8). In addition, adhesion molecules can present a similar behavior involving tumoral zones and AE (15), indicating that molecular evaluation of AE may provide information concerning tumor behavior (6). Our results suggest that the more advanced pTNM stages correspond to higher MIIB immunoreactivity in AE (*p* = 0.057). Considering that tumors such as melanoma, with high potential for tissue invasion and metastasis, express high levels of MIIB (16), it is possible to suggest detection of MIIB expression in AE as a potential sign of regional metastasis. Myosin IIB may contribute to cancer cell invasion by maintaining cell contractility, which is required for their passage through pores in the extracellular matrix (13,17).

Traditionally, in 80-90% of patients suffering from HNSCC, the disease is related to alcohol and tobacco exposure (18). A possible challenge to this assumption can be made based on the present sample since there were a number of patients who stopped their habits long before development of the disease. This decrease in the number of patients exposed to risk factors for HNSCC may be attributed to new initiatives in public health targeting the prevention of HNSCC and related illnesses (19). Nevertheless, we have to consider that an important number of our sample (31.5%) is of the neck region, and that, 70.4% of the sample was composed by younger patients (below 65 years). Since HPV is related to the pathogenesis of the disease mostly in the oropharynx in younger patients (1), our data suggest, that HNSCC development may have HPV as an etiological factor. Further studies are needed to define the role of HPV in HNSCC carcinogenesis; however, it is possible that interactions between HPV oncoproteins and cell cycle mediators lead to cell cycle progression, cell immortalization, apoptosis suppression and essentially decoupling of proliferation and maturation, causing the HNSCC (1).

A correlation was observed between immunostaining for MIIA (*p* = 0.048) and MIIB (*p* = 0.010) in AE in patients exposed to alcohol for periods longer than 10 years. According to studies of the relationship between alcohol consumption and HNSCC, it was observed that chronic alcohol consumption can cause a reduction in epithelial thickness and consequently an increase in cellular desquamation, causing an adaptive response of the epithelium and increasing the proliferation of the basal coat (20) and the possibility of errors during DNA duplication. Additionally, proto-oncogenes as well as tumor suppressor genes, which are altered during alcohol exposure, may influence the expression and/or regulation of MII isoform activity (21,22). Considering that the damage caused by alcohol is related to the duration of exposure (20,23), since the cell has DNA damage and acquires tumoral behavior, it can assume a migratory phenotype by activation of MIIA and MIIB (24).

During cellular migration, there is cooperation between MIIA and MIIB because during the protrusion of the cellular body, MIIA is responsible for adhesion between cells and the extracellular environment, whereas MIIB is responsible for stabilization of adhesion complexes. This synergy may justify the fact that in the tumoral zones, increased MIIA expression is related to increased MIIB expression (*p* = 0.001) (25).

The present study demonstrated the presence of MIIA in both AE and tumoral zones. However, it does not correlate this presence with either clinical staging or clinical outcome. Even so, it is important to note that the literature contains paradoxical findings regarding this protein (17). In pulmonary adenocarcinoma, it was observed that patients who did not present immunoreactivity to MIIA had no cancer recurrence for 5 years, concluding that non-expression of MIIA is a predictive factor for good evolution, in agreement with other studies involving esophageal and stomach carcinomas (26,27). These studies appear to contradict the reports by Schramek *et al.* (28) and Conti *et al.* (29) that state that loss of MIIA appeared to be related to tumor initiation.

MIIC is one of the proteins responsible for breaking intercellular bridges present during final cell division. Defects in this process may result in binucleated cells and alterations in the expression and/or activity of this protein, generating abnormal cytokinesis, which could foster the propagation of genetic defects such as aneuploidy (30,31) in breast and pulmonary cancer cell lines. Takaoka *et al.* suggested that formation of the contractile ring at the last step of cell division depends on MIIC as a critical stage in cytokinesis, offering an explanation for how chromosomal instability may arise in breast cancer (32). This study showed that there was a worsening of the prognosis of patients whose tumors expressed MIIC (*p* = 0.048) (Fig. 2) and that negative evolution in patients was correlated to increased MIIC in the ZI (*p* = 0.017). Since tumoral progression occurs in the ZI, and because the physical forces generated by MIIC are necessary for cell division, we can suggest that MIIC expression in the ZI is a predictive factor for the negative evolution of HNSCC patients in cases where the cause of clinical failure is tissue invasion (4).

Although the role of MIIs in HNSCC progression is still not clear, the present work suggests that MIIB expression in AE may indicate the potential for regional metastasis and that MIIC expression in the tumoral ZI is predictive of negative evolution of the disease.
